# The Immunologic Paradox of BTK Inhibitors in Chronic Lymphocytic Leukemia: Selectivity, Hypogammaglobulinemia, and Infection Risk

**DOI:** 10.3390/cancers18101621

**Published:** 2026-05-17

**Authors:** Mihaela Andreescu, Sorin Ioan Tudorache, Cosmin Alec Moldovan, Adina-Diana Moldovan, Daniel Cochior, Viola Popov, Bogdan Andreescu, Diana Ionescu, Monica-Daniela Padurariu-Covit

**Affiliations:** 1 Department of Medical-Surgical and Prophylactic Disciplines, Faculty of Medicine, Titu Maiorescu University of Bucharest, 031593 Bucharest, Romania; tevetmihaela@gmail.com (M.A.); daniel.cochior@prof.utm.ro (D.C.); dianaionescu@yahoo.com (D.I.); 2 Department of Hematology, Colentina Clinical Hospital, 020125 Bucharest, Romania; violamariap@gmail.com; 3 Department of Preclinical Disciplines, Faculty of Medicine, Titu Maiorescu University, 031593 Bucharest, Romania; sorin.tudorache@prof.utm.ro (S.I.T.); diana.moldovan@prof.utm.ro (A.-D.M.); 4 Department of General Surgery, Witting Clinical Hospital, 010243 Bucharest, Romania; 5 Medical Doctoral School, Titu Mariorescu University, 040317 Bucharest, Romania; 6 Department of General Surgery, Monza Clinical Hospital, 021968 Bucharest, Romania; 7 Department of Plastic Surgery, Colentina Clinical Hospital, 020125 Bucharest, Romania; doctorandreescu@yahoo.com; 8 Department of ENT, Gomoiu Clinical Hospital, 022102 Bucharest, Romania; 9 Research Centre in the Medical-Pharmaceutical Field, Faculty of Medicine and Pharmacy Dunarea de Jos University, 800008 Galati, Romania; monica.padurariu@ugal.ro; 10 Sf. Apostol Andrei County Emergency Clinical Hospital, 800578 Galati, Romania

**Keywords:** BTK inhibitors, Ibrutinib, Acalabrutinib, Pirtobrutinib, hypogammaglobulinemia, infection risk, T-cell immunity, chronic lymphocytic leukemia, B-cell malignancies, immunomodulation

## Abstract

Bruton’s tyrosine kinase (BTK) inhibitors have revolutionized B-cell malignancy treatment but paradoxically increase infection susceptibility. Covalent BTK inhibitors (Ibrutinib, Acalabrutinib, Zanubrutinib) induce sustained BTK blockade but disrupt immune homeostasis through off-target effects on T-cell and myeloid signaling, contributing to hypogammaglobulinemia and increased risk of bacterial, viral, and opportunistic fungal infections. Non-covalent inhibitors (Pirtobrutinib) and emerging BTK degraders offer more selective inhibition, preserving T-cell function and potentially mitigating infection risk, though their long-term immunological impact requires further study. Infection susceptibility varies across BTK inhibitor generations, reflecting differences in kinase selectivity, modulation of humoral and cellular immunity, and disease-intrinsic immune dysfunction in chronic lymphocytic leukemia. This review examines the mechanistic basis of BTK inhibitor-associated immune dysfunction, compares generational differences in selectivity and safety profiles, and provides evidence-based recommendations for infection risk mitigation in clinical practice.

## Simple Summary

Bruton’s tyrosine kinase inhibitors have transformed treatment for B cell cancers but can unexpectedly raise infection risk by lowering protective antibodies and changing immune cells, especially T cells. This review explains how older, less selective drugs cause broader immune disruption while newer, more selective and reversible agents preserve T cell function and show lower infection rates. We compare drug generations, summarize clinical evidence linking falling antibody levels to infections, and outline practical steps clinicians can take to reduce risk such as regular immune monitoring, choosing more selective agents for vulnerable patients, vaccination, and using antibody replacement when needed. By clarifying the mechanisms behind immune changes and highlighting safer treatment choices, this work aims to help doctors and researchers balance cancer control with better protection from infections.

## 1. Introduction

Bruton’s tyrosine kinase (BTK) inhibitors have emerged as a transformative class of targeted therapies for B-cell malignancies and autoimmune diseases [1]. Agents such as Ibrutinib and Acalabrutinib covalently bind to the cysteine-481 residue of BTK, thereby inhibiting its kinase activity and disrupting B-cell receptor (BCR) signaling pathways critical for the survival and proliferation of malignant B cells [2]. Ibrutinib was approved in 2016 by U.S. Food and Drug Administration (FDA) for first-line treatment of chronic lymphocytic leukemia (CLL), owing to its durable efficacy and manageable safety profile [3]. However, its broad off-target effects, including inhibition of interleukin-2-inducible T-cell kinase (ITK) and epidermal growth factor receptor (EGFR), raised concerns about toxicity. This prompted the development of more selective next-generation inhibitors like Acalabrutinib, which retain efficacy while minimizing off-target activity. Acalabrutinib has demonstrated particular benefit in patients with high-risk cytogenetic abnormalities, such as deletion of chromosome 17p13.1 [4].

In 2021, data from randomized phase III trials, including the ELEVATE-TN study, confirmed that Acalabrutinib is non-inferior to Ibrutinib in relapsed or treatment-naive CLL. These findings support the use of Acalabrutinib either as monotherapy or in combination with obinutuzumab as a frontline option for symptomatic CLL [5,6]. More recently, the development of reversible (non-covalent) BTK inhibitors, such as Pirtobrutinib, has further advanced the field. These agents are designed to overcome resistance mutations like BTK C481S, which impair the binding of covalent inhibitors. Pirtobrutinib demonstrates high selectivity and activity against both wild-type and mutant BTK, with the BRUIN trial showing overall response rates of 82% in covalent BTK inhibitor-pretreated CLL patients and median progression-free survival of 19.4 months [7,8]. BTK is essential for B-cell maturation, antibody synthesis, and Fcγ receptor signaling in myeloid cells. Additionally, BTK suppression affects Toll-like receptor (TLR) signaling, and B cell adhesion and migration, as well as cells inside the tumor microenvironment [9].

Infections represent a well-recognized cause of morbidity and mortality among patients with CLL, particularly during treatment [10]. BTK inhibitors have been associated with an increased incidence of bacterial infections, most notably respiratory tract infections, as well as opportunistic and invasive fungal infections [11]. Invasive fungal infections caused by Aspergillus species have been documented most commonly within the first six months of treatment [12]. A meta-analysis encompassing 12 studies found that BTK inhibitors significantly increased the risk of upper respiratory tract infections in patients with B-cell malignancies, with a 1.55-fold higher incidence compared to control groups [13]. Next-generation BTK inhibitors demonstrate more favorable safety profiles; Zanubrutinib shows decreased infection frequency, lower atrial fibrillation risk, and enhanced progression-free survival compared to Ibrutinib in the ALPINE trial [14]. This review examines the relationship between BTK inhibitor therapy, alterations in immunoglobulin levels, and susceptibility to infections, assessing the influence of differing kinase selectivity on immune modulation and highlighting clinical strategies to mitigate infection risk.

## 2. Literature Search Strategy

**Figure 1:**
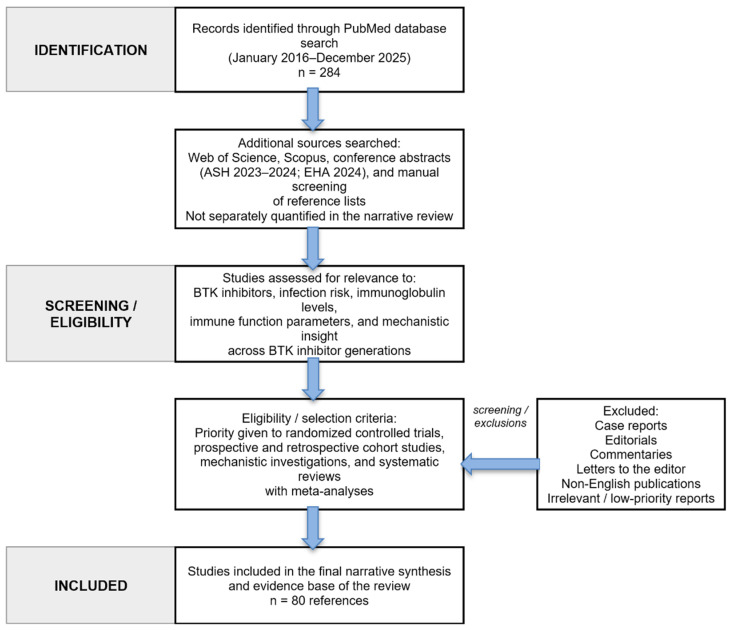
Modified PRISMA-style flow diagram of the literature search and study selection process. The diagram summarizes database searching, additional source screening, eligibility assessment, and final inclusion for the narrative review of BTK inhibitors, immunoglobulin changes, infection risk, and T-cell immunity in chronic lymphocytic leukemia.

A comprehensive literature search was conducted using PubMed, Web of Science, and Scopus databases covering publications from January 2016 to December 2025. Search terms included “BTK inhibitor,” “Ibrutinib,” “Acalabrutinib,” “Zanubrutinib,” “Pirtobrutinib,” combined with “infection,” “hypogammaglobulinemia,” “immunoglobulin,” “T-cell immunity,” and “chronic lymphocytic leukemia.” Boolean operators (AND, OR) were used to combine search terms systematically. As this work represents a narrative review, study selection was guided by clinical relevance, mechanistic insight, and evidence informing infection risk and immune modulation across BTK inhibitor generations (Figure 1). Priority was given to randomized controlled trials, prospective and retrospective cohort studies, mechanistic investigations, and systematic reviews with meta-analyses. Conference abstracts were also considered to capture emerging data. Case reports, editorials, commentaries, letters, and non-English publications were excluded. Overall, 80 references were included, reflecting both systematic database retrieval and targeted manual inclusion to capture emerging therapeutic modalities and immunological findings.

## 3. Mechanism of Action of BTK Inhibitors

**Figure 2:**
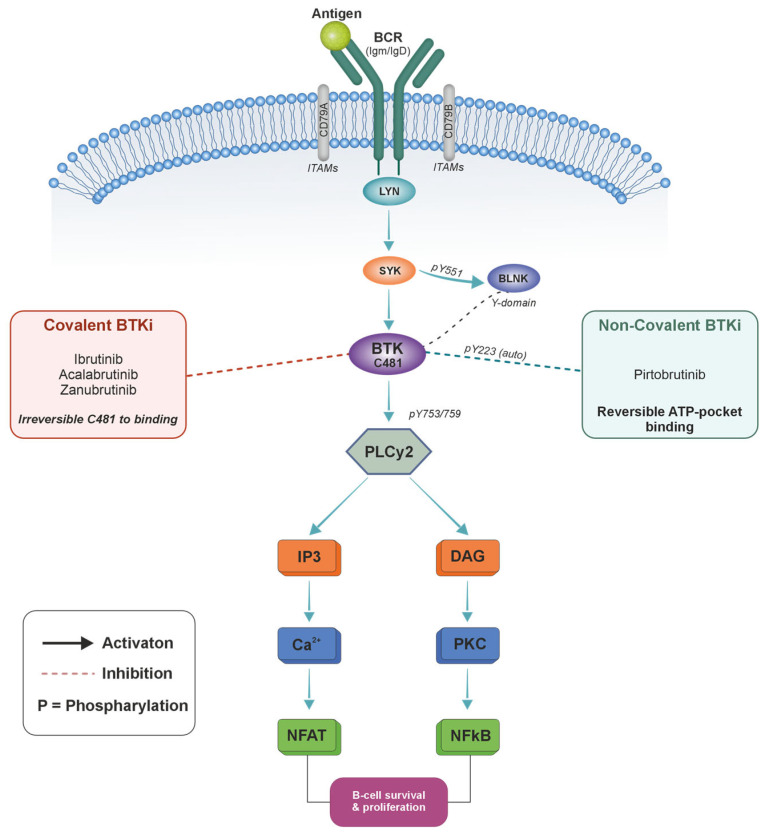
B-Cell Receptor Signaling Cascade and BTK Inhibitor Mechanisms. Upon antigen binding to the B-cell receptor (BCR), receptor clustering triggers LYN-mediated phosphorylation of CD79a/b immunoreceptor tyrosine-based activation motifs (ITAMs), recruiting spleen tyrosine kinase (SYK). SYK phosphorylates B-cell linker protein (BLNK), creating docking sites for downstream effectors. Bruton’s tyrosine kinase (BTK) is recruited via its pleckstrin homology (PH) domain, phosphorylated by SYK at tyrosine 551 (Y551), and subsequently autophosphorylates at tyrosine 223 (Y223) for full activation. Activated BTK phosphorylates PLCγ2 at Y753 and Y759, generating second messengers inositol trisphosphate (IP3) and diacylglycerol (DAG). IP3 triggers calcium mobilization and nuclear factor of activated T-cells (NFAT) activation, while DAG activates protein kinase C (PKC) and the nuclear factor kappa B (NF-kB) pathway. These converging pathways drive B-cell survival and proliferation. Covalent BTK inhibitors (Ibrutinib, Acalabrutinib, Zanubrutinib) irreversibly bind to the cysteine-481 (C481) residue in the BTK active site, providing sustained kinase inhibition. Non-covalent BTK inhibitors (Pirtobrutinib) reversibly occupy the ATP-binding pocket, maintaining activity against C481S-mutant BTK and enabling potential immunoglobulin recovery through intermittent inhibition. Conceptual illustration based on data from [3,9].

BTK belongs to the TEC family of non-receptor tyrosine kinases, which includes five members: BTK, TEC, ITK, RLK/TXK, and BMX. These kinases share structural homology and play key roles in hematopoietic cell signaling. BTK is a critical mediator of B-cell receptor (BCR) signaling, playing a central role in B-cell development, survival, and activation. Upon BCR engagement, BTK is phosphorylated at Y551 by SYK, followed by autophosphorylation at Y223, subsequently activating downstream signaling cascades including the NF-κB and MAPK pathways, which are essential for B-cell survival and function [9]. The critical substrate of activated BTK is phospholipase Cγ2 (PLCγ2), which BTK phosphorylates at Y753 and Y759, generating second messengers IP3 and DAG that activate calcium mobilization and protein kinase C pathways (Figure 2). Inhibition of BTK disrupts these survival signals, leading to apoptosis of malignant B cells. First-generation BTK inhibitor ibrutinib is a covalent BTK inhibitor that irreversibly binds Cys-481 in the BTK active site, effectively blocking B-cell receptor signaling. In addition to BTK, ibrutinib exhibits off-target inhibition of interleukin-2-inducible T-cell kinase (ITK) and epidermal growth factor receptor (EGFR), mechanisms that contribute to its immunomodulatory profile, including alterations in T-cell subsets and potential cardiovascular effects [15]. Second-generation inhibitors, such as Acalabrutinib (ITK IC50 > 1000 nM) and Zanubrutinib, demonstrate increased selectivity for BTK, reducing interactions with non-BTK kinases and enhancing safety profiles [2]. Table 1 summarizes the comparative characteristics of BTK inhibitors across generations.

Pirtobrutinib represents a mechanistic breakthrough as a highly selective non-covalent BTK inhibitor. Rather than forming covalent bonds, Pirtobrutinib establishes an extensive hydrogen bonding network with the BTK hinge region and achieves an exceptionally long residence time of 2.4 h for wild-type BTK and 1.5 h for C481S-mutant BTK [8]. This unique ability to stabilize BTK in a closed, inactive conformation provides sustained inhibition while preserving selectivity over 363 other kinases tested.

BTK also plays an important role in myeloid cell function, particularly in Fc gamma receptor (FcγR) signaling in macrophages. Inhibition of BTK in these cells impairs antibody-dependent cellular phagocytosis, which may affect pathogen clearance [16]. The immunological consequences of differential ITK inhibition by BTK inhibitors, particularly with respect to T-cell-mediated infection protection, are discussed in detail in Section 6.

## 4. Immunoglobulin Levels and BTK Inhibitor Therapy

BTK inhibitors are associated with a gradual decline in immunoglobulin (Ig) levels, particularly IgG, primarily due to impaired plasma cell viability and disruption of BCR-mediated signaling [17]. This hypogammaglobulinemia is mechanistically linked to BTK’s role in activating NF-κB, a pathway essential for plasma cell survival. Inhibition of BTK reduces IL-6-dependent support for plasma cells, thereby promoting apoptosis [18]. Beyond direct effects on B cells and plasma cell viability, BTK inhibitors also modulate the bone marrow microenvironment that supports long-lived plasma cells. Disruption of stromal cell signaling, cytokine networks, and extracellular matrix interactions may impair plasma cell survival and antibody production, further contributing to hypogammaglobulinemia [17]. Preclinical studies have shown that Ibrutinib leads to depletion of marginal zone B-cells, which are key contributors to T-independent IgM production [19]. Longitudinal studies reveal BTK inhibitors preserve preexisting antigen-experienced B cells while impairing de novo naïve B-cell generation, maintaining the ratio of antigen-experienced to antigen-naïve B cells at approximately 39% through 24 months of therapy [20]. The extent of immunoglobulin decline is also influenced by patient-specific factors. For instance, preexisting immunodeficiency in CLL, such as low baseline IgG, strongly predicts the development of severe and persistent hypogammaglobulinemia [21]. Second-generation BTK inhibitors appear to cause less IgG reduction than Ibrutinib, possibly due to preserved T-cell production of IL-21, which supports residual plasma cell function and mitigates immunoglobulin loss [20]. Additionally, genetic mutations in BTK (e.g., C481S) or downstream effectors like PLCγ2 can alter BCR signaling dynamics, contributing to drug resistance and potentially exacerbating immune dysregulation [22]. Interestingly, clinical infection risk does not always correlate with IgG levels, reflecting broader immune dysregulation involving cellular and innate immune compartments in patients receiving BTK inhibitors [17,23,24].

## 5. Infection Risk Associated with BTK Inhibitors

BTK inhibitors markedly enhance vulnerability to infections owing to the synergistic effects of B-cell dysfunction (hypogammaglobulinemia) and unintended inhibition of T-cells or myeloid cells, resulting in compromised immunological responses [17]. Ibrutinib has been associated with a higher incidence of serious infections and substantial discontinuation rates in real-world settings, ranging from 15% to 43% [25]. In a well-characterized regional cohort of patients with CLL, 63.6% of those receiving Ibrutinib experienced at least one infection either during or following treatment [26]. A phase 2 study reported the most common grade ≥ 3 infections as pneumonia (14.5%) and urinary tract infections (5%). Long-term follow-up from the RESONATE trial confirmed the sustained efficacy and manageable safety profile of Ibrutinib, though overall infection rates were higher in the Ibrutinib group (70%) compared to the ofatumumab group (54%), with neutropenia, pneumonia, and sinusitis more frequently observed [27,28]. Table 2 summarizes infection rates from major clinical trials.

Invasive fungal infections, predominantly caused by Aspergillus and Cryptococcus species, are rare but frequently fatal, with peak incidence occurring within six months of treatment initiation [20]. A multicenter retrospective analysis reported a 23% incidence of serious infections over a 17-month period, with bacterial pathogens accounting for 54% of cases. Invasive fungal infections were observed in 6% of patients, predominantly among individuals with CLL undergoing treatment with Ibrutinib [29]. Although relatively uncommon, fungal infections remain a serious concern in patients receiving prolonged BTK inhibitor therapy. Cases of invasive aspergillosis and Pneumocystis jirovecii pneumonia have been documented, particularly in those with concurrent corticosteroid use or prolonged lymphopenia [30]. In patients with CLL treated with Ibrutinib, invasive fungal infections range from 0% to 11% in most studies [31]. Viral reactivations, including hepatitis B virus (HBV) and cytomegalovirus (CMV), have been reported during Ibrutinib therapy and are thought to reflect BTK inhibitor-associated alterations in T-cell-mediated antiviral responses, including effects related to ITK inhibition [32,33]. BTK inhibition also impairs vaccine immunogenicity; CLL patients receiving BTK inhibitors show diminished responses to recombinant zoster vaccine, suggesting compromised vaccine efficacy [34].

It is important to acknowledge that infection susceptibility in CLL reflects both disease-intrinsic immune dysfunction and treatment-related immunomodulation. Nevertheless, comparative trial data and real-world studies consistently demonstrate differential infection profiles across BTK inhibitor generations, supporting a clinically relevant drug-specific contribution beyond baseline risk. Recent reviews have emphasized that infection risk associated with targeted therapies in hematological malignancies reflects the combined impact of underlying disease-related immune dysfunction and therapy-specific immunomodulatory effects [35].

## 6. BTK Inhibitor Selectivity and T-Cell-Mediated Immune Modulation

BTK inhibitors differentially influence T-cell activity based on their kinase selectivity [36]. Conversely, second-generation inhibitors such as Acalabrutinib do not block ITK, hence maintaining CD8+ cytotoxic T-cell functionality and interferon-γ (IFN-γ) synthesis, which reduces the risk of infection [37]. BTK inhibition also disrupts B-cell and T-cell interaction by decreasing mitochondrial respiration in circulating B-cells, inhibiting activation-induced production of costimulatory molecules, and inducing an anti-inflammatory shift in B-cell responses, which correlates with a reduction in T-cell pro-inflammatory responses [38]. Furthermore, BTK inhibition reduces B-cell synthesis of cytokines such as IL-10, which typically facilitate regulatory T-cell (Treg) functionality, potentially exacerbating immunological dysregulation [39].

Compensatory T-cell mechanisms may mitigate humoral deficiencies. In patients treated with BTK inhibitors, CD8+ T-cells demonstrate elevated perforin and granzyme expression, facilitating pathogen clearance despite hypogammaglobulinemia. These observations highlight the dual function of T-cells in both mediating and compensating for the immunosuppression generated by BTK inhibitors [1]. Additionally, Ibrutinib has been shown to reduce the immunosuppressive features of CLL cells through both BTK-dependent and BTK-independent mechanisms, potentially improving T-cell function over time [40]. The broader downstream impact of BTK inhibition includes modulation of the NF-κB and MAPK pathways, which are essential not only for B-cell survival and proliferation but also for regulating inflammatory cytokine production and overall immune responses [41].

Differences in kinase selectivity among BTK inhibitors critically shape this balance [42,43]. The C481S resistance mutation, detected in approximately 37% of Ibrutinib-progressing patients and representing 91% of all BTK mutations, eliminates the nucleophile required for covalent bond formation [44]. The susceptibility of compound variants to Ibrutinib suggests that mutations with smaller side chains are less prone to steric interference with adjacent residues [45]. Covalent BTK inhibitors also exhibit broader kinase inhibition profiles, including off-target activity against ITK, which contributes to their distinct immunological safety profiles [15].

**Figure 3:**
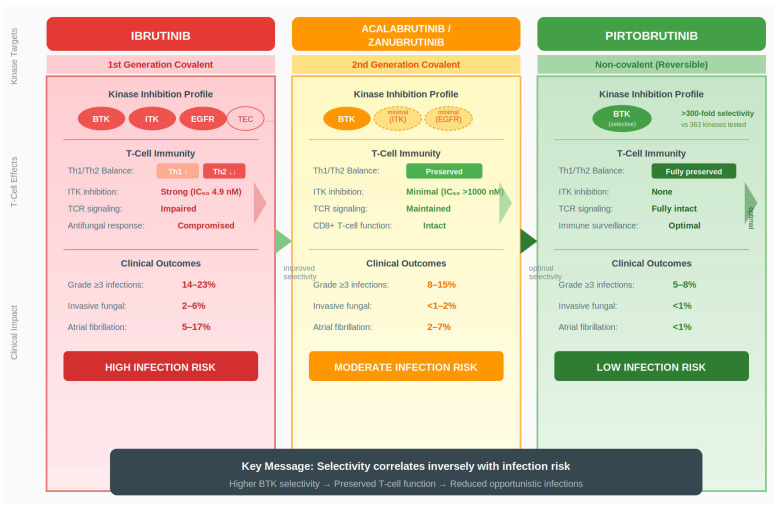
Differential Immunological Effects of Covalent versus Non-covalent BTK Inhibitors. Comparative analysis of kinase selectivity profiles, T-cell immunity effects, and clinical outcomes across three generations of BTK inhibitors. First-generation covalent inhibitors (Ibrutinib) demonstrate broad kinase inhibition including BTK, interleukin-2-inducible T-cell kinase (ITK; IC50 4.9 nM), epidermal growth factor receptor (EGFR), and TEC family kinases. This off-target activity disrupts T-helper cell balance (decreased Th2, relatively increased Th1), impairs T-cell receptor (TCR) signaling, and compromises antifungal immunity. Clinical consequences include grade ≥ 3 infections (14–23%), invasive fungal infections (2–6%), and atrial fibrillation (5–17%), representing high infection risk. Second-generation covalent inhibitors (Acalabrutinib, Zanubrutinib) exhibit enhanced BTK selectivity with minimal ITK inhibition (IC50 >1000 nM for Acalabrutinib). This selectivity preserves Th1/Th2 balance, maintains TCR signaling, and keeps CD8+ T-cell function intact. Improved clinical outcomes include reduced grade ≥ 3 infections (8–15%), lower invasive fungal infection rates (<1–2%), and decreased atrial fibrillation (2–7%), representing moderate infection risk. Non-covalent reversible inhibitors (Pirtobrutinib) achieve exceptional selectivity (>300-fold over 363 kinases tested) with no ITK inhibition. T-cell immunity is fully preserved with optimal immune surveillance. Clinical safety profile shows grade ≥ 3 infections (5–8%), invasive fungal infections (<1%), and atrial fibrillation (<1%), representing low infection risk. The key message demonstrates that higher BTK selectivity correlates inversely with infection risk through preserved T-cell function and reduced opportunistic infections. Conceptual illustration based on data from [15,17,42,46,47].

Non-covalent BTK inhibitors demonstrate reversible BTK inhibition, which may facilitate partial immunoglobulin recovery and potentially reduce infection risks linked to extended immunosuppression [46]. Second-generation covalent inhibitors (Acalabrutinib, Zanubrutinib) achieve improved BTK selectivity, minimal ITK inhibition, and preserved Th1/Th2 balance, reducing direct T-cell suppression and supporting immune restoration [37]. Clinically, these differences translate into differential safety outcomes: first-generation inhibitors are associated with grade ≥ 3 infections in 14–23% of patients, whereas second-generation covalent and non-covalent inhibitors show lower rates (8–15% and 5–8%, respectively) and reduced opportunistic infections, illustrating that higher selectivity maximizes the protective immune balance [17,46]. Resistance mutations further influence this landscape. The BTK C481S mutation prevents covalent inhibitor binding, while gatekeeper mutations such as T474I confer resistance to non-covalent inhibitors [44,47]. Compound mutations combining C481S and T474I create super-resistant variants, emphasizing the ongoing need for therapies that maintain both tumor control and immune preservation [45]. Differences in kinase selectivity between covalent and non-covalent BTK inhibitors translate into distinct effects on immune surveillance, particularly through modulation of T-cell function (Figure 3), as discussed in Section 6.

## 7. Clinical Strategies to Mitigate Infection Risk

It is essential to monitor immunoglobulin levels and immunological function in patients undergoing treatment with BTK inhibitors, since these medications may block B-cell responses, resulting in hypogammaglobulinemia and heightened vulnerability to infections. Repeated assessments of IgG, IgA, and IgM facilitate early identification and intervention [20]. Immunoglobulin replacement therapy follows guideline-based criteria: IgG below 500–600 mg/dL plus history of severe infection in the preceding 6 months or recurrent infections despite appropriate prophylaxis [48]. Infection risk stratification employs validated scoring systems; the 2024 Murru score incorporates age, IGHV mutational status, immunoglobulin levels, and Binet stage, stratifying patients into low (7%), intermediate (14%), and high (40%) 5-year infection risk categories [21]. Combined antibody deficiency (low IgG, IgA, and IgM) associates with highest infection risk, with the number of immunoglobulin class deficits directly correlating with time to first infection [21]. Functional immunological testing, including complete lymphocyte subset analysis and vaccination response assessment, enhances personalized infection risk classification [48].

Patient selection should emphasize comorbidities and immunological state. Vulnerable patients or those with advanced disease may benefit from non-covalent BTK inhibitors (e.g., Pirtobrutinib), which have fewer off-target immunosuppressive effects [49]. Personalized medication modifications, including dosage optimization and strategically scheduled treatment interruptions during active infections, assist in reducing infection risks without markedly undermining therapeutic effectiveness [50]. Antimicrobial prophylaxis should be risk-stratified: Pneumocystis prophylaxis is not routinely recommended for BTK inhibitor monotherapy but should be considered in high-risk patients with relapsed/refractory disease, concurrent corticosteroids, or prior opportunistic infections. Hepatitis B screening is mandatory, with prophylaxis required for HBsAg-positive patients [30]. Combination therapies with BCL-2 inhibitors show promise in reducing continuous BTK inhibitor exposure; the FLAIR trial demonstrated 97% progression-free survival at 3 years with Ibrutinib–venetoclax, while CAPTIVATE showed 90% progression-free survival at 5 years with fixed-duration therapy achieving over 70% undetectable minimal residual disease [51,52]. However, infection risk remains significant during active therapy. In the FLAIR trial, infections were the most frequent serious adverse events, occurring in 47.3% of patients during treatment while rates decreased after treatment cessation [51]. Similarly, the GLOW trial evaluating fixed-duration ibrutinib-venetoclax in treatment-naïve CLL, reported that ibrutinib–venetoclax combination achieved a 42-month progression-free survival rate of 74.6% and reduced the risk of disease progression or death by nearly 79% compared with chlorambucil–obinutuzumab (HR 0.214, 95% CI 0.138–0.334; *p* < 0.0001). The regimen also demonstrated a manageable safety profile, with few treatment-related deaths and fewer post-treatment infection-related deaths [53]. These findings underscore that although fixed-duration therapy may reduce long-term immunologic exposure, infection monitoring and prophylaxis remain critical.

## 8. Future Directions and Research Perspectives

The balance between BTK inhibitor efficacy and immunological preservation remains a critical research focus. Development of highly selective inhibitors aims to diminish off-target effects, thereby reducing immunosuppression and infection risks. Protein degraders (PROTACs) represent the next frontier, inducing ubiquitination and proteasomal degradation of BTK rather than simply inhibiting kinase activity [54,55].

### 8.1. BTK Protein Degraders: A Mechanistic Leap Beyond Inhibition

PROTACs simultaneously bind BTK and an E3 ubiquitin ligase (predominantly CRBN), triggering degradation through proximity-driven ubiquitination [56,57]. Critically, because PROTACs act catalytically and are recycled after each degradation event, they do not require sustained target occupancy—an advantage with direct implications for overcoming resistance mutations such as C481S, which impair covalent inhibitor binding, and T474I, which neutralizes Pirtobrutinib [58]. Four BTK degraders are currently in active clinical development: BGB-16673, NX-2127, NX-5948, and AC-676, each with distinct selectivity profiles and immunomodulatory properties [42].

### 8.2. BGB-16673 (CaDAnCe-101)

BGB-16673 is the most clinically advanced BTK degrader, having received FDA fast track designation for adults with relapsed/refractory CLL or SLL following two or more prior lines of treatment including a BTK inhibitor and a BCL-2 inhibitor [59]. The agent induces degradation of both wild-type BTK and resistance-conferring mutants through cereblon-mediated ubiquitination, with a half-life of 7.2–10 h enabling once-daily oral administration [60]. In the phase 1/2 CaDAnCe-101 trial, among 49 response-evaluable patients with CLL/SLL, the overall response rate was 77.6%, rising to 93.8% at the 200 mg dose level, with responses observed even at the lowest dose of 50 mg [61]. Updated results presented at EHA 2025, with a median follow-up of 15.6 months, confirmed an overall response rate of 93.8% at the 200 mg dose, with a generally tolerable safety profile and no unexpected toxicities [62]. Notably, the agent has demonstrated activity in Richter transformation, a population historically associated with poor outcomes, as well as in other B-cell malignancies including Waldenström macroglobulinemia [63].

A phase 3 randomized trial comparing BGB-16673 against investigator’s choice in patients with prior BTK and BCL-2 inhibitor exposure is underway, with progression-free survival at 36 months as the primary endpoint [55]. From an immunological standpoint, grade 3 or higher adverse events included neutrophil count decreases (20%) and pneumonia (12%), reflecting the vulnerability of a heavily pretreated, immunocompromised population [64,65].

### 8.3. NX-2127: A Dual-Function Degrader with Immunomodulatory Activity

NX-2127 represents a mechanistically unique approach: in addition to inducing BTK degradation, it concurrently degrades the CRBN neosubstrates Ikaros (IKZF1) and Aiolos (IKZF3) through its thalidomide-based IMiD moiety. In preclinical studies using primary CLL cells, NX-2127 significantly upregulated IFN-γ and IL-2 under Th1-polarizing conditions to a degree exceeding the effect of either lenalidomide or Ibrutinib, without altering Th2 differentiation markers [66]. Furthermore, NX-2127 and lenalidomide, but not NX-5948 or Ibrutinib, enhanced T-cell-mediated killing of NHL cells in cytotoxicity assays, accompanied by increased granzyme B secretion by CD8+ T cells, and promoted immunological synapse formation to levels comparable to lenalidomide [67]. These findings suggest that NX-2127’s dual mechanism may partially counteract CLL-associated T-cell exhaustion [68].

### 8.4. NX-5948: Selective BTK Degradation with CNS Penetration

NX-5948 is a highly selective CRBN-recruiting BTK degrader that lacks IMiD neosubstrate activity, providing a cleaner dissection of the effects of BTK protein elimination from immunomodulatory side effects. Preclinical data demonstrated potent and rapid BTK degradation in primary human B cells (DC50 = 0.034 nM at 4 h), with deep suppression of BCR-, TLR-, and FcR-mediated activation in both B cells and myeloid cells at sub-nanomolar potency [69]. A unique feature is its demonstrated capacity to cross the blood–brain barrier in animal models, suggesting potential applications in CNS lymphoma. In the phase 1 trial (NCT05131022), NX-5948 achieved an overall response rate of 69.2% in CLL patients, with all responses ongoing as of the April 2024 data cut, in a heavily pretreated population with unfavorable genetic mutations [67]. Importantly, unlike NX-2127, NX-5948 did not modulate T-cell activation markers, Treg differentiation, or immunological synapse formation, indicating that the T-cell immunomodulatory effects observed with NX-2127 are specifically dependent on CRBN neosubstrate activity rather than BTK degradation per se [70]. This distinction has direct implications for understanding the relative infection risk profiles of agents within this class [71].

### 8.5. AC-676 and Emerging Degraders

AC-676, developed by Accutar Biotech using a protein–protein interaction targeting chimera (PPI-TAC) platform, is a fourth BTK degrader currently in phase 1 dose escalation (NCT05780034), linking a BTK-binding moiety to a cereblon E3-ligase recruiting ligand to induce BTK ubiquitination and proteasomal degradation, with preliminary safety and efficacy data expected in 2025 [72].

## 9. Immunological Implications of Complete BTK Elimination

The immunological consequences of full BTK protein degradation extend beyond the BCR signaling axis [73]. BTK’s scaffolding role in TLR signaling and its involvement in Fcγ receptor-mediated phagocytosis in macrophages suggest that complete protein removal could exert broader effects on innate immune responses than kinase inhibition alone. Preclinical data confirm that NX-5948 achieves equal or greater suppression of TLR- and FcR-mediated myeloid cell activation compared to BTK inhibitors [74], raising the theoretical concern that degraders might further impair macrophage-mediated fungal clearance—the mechanism underlying invasive aspergillosis risk with Ibrutinib [75]. However, the absence of ITK off-target activity in all four clinical degraders preserves T-cell receptor signaling, potentially mitigating the antifungal immunity deficits most characteristic of first-generation BTK inhibitors [15]. Prospective immunological monitoring within ongoing degrader trials—including comprehensive lymphocyte subset analysis, immunoglobulin kinetics, and vaccine response assessment—will be essential to characterize the net infectious risk of this emerging class [34].

Combination strategies continue evolving to improve efficacy while enabling time-limited therapy [76]. Triple combinations (BTK inhibitor plus venetoclax plus anti-CD20 antibody) achieve unprecedented depth of response, with the BOVen regimen (Zanubrutinib-venetoclax-obinutuzumab) demonstrating 92% bone marrow undetectable MRD at 5-year follow-up [77]. MRD-guided treatment duration strategies may optimize individual patient outcomes while minimizing cumulative immunosuppression. Unresolved questions persist regarding memory B-cell reconstitution, vaccine responsiveness during and after therapy, and genetic determinants of immunoglobulin decline [78]. Integration of biomarkers including β2-microglobulin kinetics and circulating tumor DNA for resistance monitoring into treatment algorithms promises to personalize therapy while maximizing benefit and minimizing immunological compromise [79].

The COVID-19 pandemic has highlighted additional infectious vulnerabilities in patients receiving BTK inhibitors. Evidence indicates that these patients often exhibit impaired seroconversion and reduced antibody responses following SARS-CoV-2 mRNA vaccination, likely due to the combined effects of CLL-associated immune dysregulation and BTK inhibitor-mediated humoral suppression [80]. Reported COVID-19 outcomes include increased severity and prolonged viral shedding, emphasizing the need for continued vigilance, risk-adapted prophylaxis, and strategies to optimize vaccine efficacy in this population [81]. Given the known impairment of vaccine immunogenicity in patients receiving BTK inhibitors, clinicians should assess vaccination status prior to initiating therapy. Where feasible, administration of recommended vaccines including influenza, pneumococcal, and SARS-CoV-2 mRNA vaccines 2–4 weeks before starting BTK inhibitor therapy allows optimal immune response [82]. Post-treatment vaccination may be less effective, particularly during active BTK inhibition or in the context of hypogammaglobulinemia. While next-generation BTK inhibitors such as Pirtobrutinib and emerging BTK degraders demonstrate lower infection risks due to enhanced selectivity or complete protein degradation, practical barriers such as cost, insurance coverage, and global availability may limit their use. In many regions, Ibrutinib remains the only accessible BTK-targeted therapy [83].

This study has several limitations. First, as a narrative literature review, it does not follow the full methodological rigor of a systematic review or meta-analysis and is therefore subject to selection and interpretative bias. Second, the included evidence is heterogeneous with respect to study design, patient populations, disease settings, duration of follow-up, and reported immunological and infectious endpoints, which limits direct cross-study comparability. Third, some emerging data discussed in this review derive from early-phase studies whose conclusions may change with longer follow-ups. In addition, although the review addresses BTK inhibitors across B-cell malignancies, much of the available evidence is derived from chronic lymphocytic leukemia, which may limit the generalizability of some conclusions to other disease contexts. Finally, the restriction to English-language publications and the narrative prioritization of clinically relevant and mechanistic studies may have introduced publication and reporting bias.

## 10. Conclusions

BTK inhibitors exhibit a paradoxical nature: they provide highly effective control of B-cell malignancies while simultaneously predisposing patients to immune dysfunction and increased susceptibility to infections. This paradox reflects the interplay between disease-intrinsic immune impairment in chronic lymphocytic leukemia and treatment-related immunomodulation. Importantly, comparative clinical trial and real-world data demonstrate that differences in kinase selectivity across BTK inhibitor generations translate into distinct immunological and infectious risk profiles, supporting a clinically relevant drug-specific contribution beyond baseline disease risk.

From a clinical standpoint, these findings emphasize the importance of individualized treatment selection, proactive monitoring of immunoglobulin levels, and risk-adapted antimicrobial prophylaxis in vulnerable patients. Fixed-duration combination regimens incorporating BCL-2 inhibitors achieve deep remissions with high rates of undetectable minimal residual disease and may limit cumulative immune toxicity compared with continuous monotherapy. Looking ahead, emerging therapeutic approaches, including reversible BTK inhibitors and BTK degraders, offer promising strategies to overcome resistance while preserving immune function. In parallel, emerging data suggest that host immune evasion mechanisms, including HLA-related factors, may further influence immune responses and clinical outcomes during targeted therapy, underscoring the need for integrative, personalized approaches in future studies. Future research should focus on immune reconstitution, vaccination strategies, and personalized treatment algorithms to optimize long-term outcomes in this rapidly evolving therapeutic landscape.

## Figures and Tables

**Table 1:** Comparative characteristics of BTK inhibitors.

Characteristic	Ibrutinib	Acalabrutinib	Zanubrutinib	Pirtobrutinib
Generation	1st (covalent)	2nd (covalent)	2nd (covalent)	Non-covalent
BTK IC50 (nM)	0.5	3	0.3	3.2
ITK IC50 (nM)	4.9	>1000	33	No inhibition
C481S activity	Lost	Lost	Lost	Preserved
Half-life (hours)	4–6	1–2	2–4	19
Atrial fibrillation	5–17%	3–7%	2–4%	<1%
Grade ≥ 3 infections	14–23%	10–15%	8–12%	5–8%

IC50: half-maximal inhibitory concentration.

**Table 2:** Infection rates associated with BTK inhibitors in clinical trials.

Trial	Agent	Population	Any Infection	Grade ≥ 3	IFI	Follow Up (Months)
RESONATE	Ibrutinib	R/R CLL	70%	24%	2–6%	65.3 (0.3–71.6)
RESONATE-17	Ibrutinib	del(17p) CLL	68%	19.5%	3%	27.6 (14.6–27.7)
ELEVATE-TN	Acalabrutinib	TN CLL	52%	14%	1%	74.5
ALPINE	Zanubrutinib	R/R CLL	48%	12%	<1%	54.2 (0.1–73.5)
BRUIN	Pirtobrutinib	cBTKi-pretreated	38%	8%	<1%	46.5 (35.5–54.7)

R/R: relapsed/refractory; TN: treatment-naïve; IFI: invasive fungal infection; cBTKi: covalent BTK inhibitor. Note: Direct comparisons of infection rates across trials should be interpreted cautiously. Confounding factors include differences in baseline immune exhaustion, median follow-up durations, and external events such as the COVID-19 pandemic, which likely influenced infection incidence in more recent trials.

## Data Availability

No new data were created or analyzed in this study. Data sharing is not applicable.

## Abbreviations

The following abbreviations are used in this manuscript:

ASH	American Society of Hematology
ATP	Adenosine triphosphate
BCL-2	B-cell lymphoma 2
BCR	B-cell receptor
BLNK	B-cell linker protein
BTK	Bruton’s tyrosine kinase
C481	Cysteine-481
cBTKi	Covalent BTK inhibitor
CD79a/b	CD79a/CD79b, signaling components of the B-cell receptor complex
CD8+	Cluster of differentiation 8-positive T cells
CLL	Chronic lymphocytic leukemia
CMV	Cytomegalovirus
CNS	Central nervous system
CRBN	Cereblon
DAG	Diacylglycerol
DC50	Half-maximal degradation concentration
E3	Ubiquitin ligase E3
EGFR	Epidermal growth factor receptor
EHA	European Hematology Association
FcR	Fc receptor
FcγR	Fc gamma receptor
FDA	U.S. Food and Drug Administration
HBsAg	Hepatitis B surface antigen
HBV	Hepatitis B virus
HLA	Human leukocyte antigen
IC50	Half-maximal inhibitory concentration
IFI	Invasive fungal infection
IFN-γ	Interferon-gamma
Ig	Immunoglobulin
IgA	Immunoglobulin A
IgG	Immunoglobulin G
IGHV	Immunoglobulin heavy-chain variable region
IgM	Immunoglobulin M
IKZF1	Ikaros family zinc finger 1 (Ikaros)
IKZF3	Ikaros family zinc finger 3 (Aiolos)
IL-10	Interleukin-10
IL-2	Interleukin-2
IL-21	Interleukin-21
IL-6	Interleukin-6
IMiD	Immunomodulatory imide drug
IP3	Inositol trisphosphate
ITAMs	Immunoreceptor tyrosine-based activation motifs
ITK	Interleukin-2-inducible T-cell kinase
LYN	LYN proto-oncogene, Src family tyrosine kinase
MAPK	Mitogen-activated protein kinase
mg/dL	Milligrams per deciliter
MRD	Minimal residual disease
NFAT	Nuclear factor of activated T-cells
NF-κB (NF-kB)	Nuclear factor kappa B
NHL	Non-Hodgkin lymphoma
nM	Nanomolar
PH	Pleckstrin homology
PKC	Protein kinase C
PLCγ2	Phospholipase C gamma 2
PPI-TAC	Protein–protein interaction targeting chimera
PROTAC	Proteolysis-targeting chimera
R/R	Relapsed/refractory
SLL	Small lymphocytic lymphoma
SYK	Spleen tyrosine kinase
TCR	T-cell receptor
Th1	T helper 1
Th2	T helper 2
TLR	Toll-like receptor
TN	Treatment-naïve
Treg	Regulatory T cell
